# Cannabinoid receptor expression in non-small cell lung cancer. Effectiveness of tetrahydrocannabinol and cannabidiol inhibiting cell proliferation and epithelial-mesenchymal transition *in vitro*

**DOI:** 10.1371/journal.pone.0228909

**Published:** 2020-02-12

**Authors:** Lara Milian, Manuel Mata, Javier Alcacer, María Oliver, María Sancho-Tello, José Javier Martín de Llano, Carlos Camps, José Galbis, Julian Carretero, Carmen Carda

**Affiliations:** 1 Department of Pathology, Faculty of Medicine and Odontology, University of Valencia, Valencia, Spain; 2 Research Institute of the University Clinical Hospital of Valencia (INCLIVA), Valencia, Spain; 3 Networking Research Center on Respiratory Diseases (CIBERER), ISCIII, Carretera Soller Bunyola, Mallorca, Illes Balears, Spain; 4 Patologika S.L. Legión Española Square, Valencia, Spain; 5 University General Hospital of Valencia, Valencia, Spain; 6 Alzira Hospital, Carretera de Corbera, Alzira, Valencia, Spain; 7 Department of Physiology, Faculty of Medicine and Odontology, University of Valencia, Valencia, Spain; Universidad Complutense de Madrid, SPAIN

## Abstract

**Background/Objective:**

Patients with non-small cell lung cancer (NSCLC) develop resistance to antitumor agents by mechanisms that involve the epithelial-to-mesenchymal transition (EMT). This necessitates the development of new complementary drugs, *e*.*g*., cannabinoid receptors (*CB1* and *CB2*) agonists including tetrahydrocannabinol (THC) and cannabidiol (CBD). The combined use of THC and CBD confers greater benefits, as CBD enhances the effects of THC and reduces its psychotropic activity. We assessed the relationship between the expression levels of *CB1* and *CB2* to the clinical features of a cohort of patients with NSCLC, and the effect of THC and CBD (individually and in combination) on proliferation, EMT and migration *in vitro* in A549, H460 and H1792 lung cancer cell lines.

**Methods:**

Expression levels of *CB1*, *CB2*, *EGFR*, *CDH1*, *CDH2* and *VIM* were evaluated by quantitative reverse transcription-polymerase chain reaction. THC and CBD (10–100 μM), individually or in combination (1:1 ratio), were used for *in vitro* assays. Cell proliferation was determined by BrdU incorporation assay. Morphological changes in the cells were visualized by phase-contrast and fluorescence microscopy. Migration was studied by scratch recolonization induced by 20 ng/ml epidermal growth factor (EGF).

**Results:**

The tumor samples were classified according to the level of expression of *CB1*, *CB2*, or both. Patients with high expression levels of *CB1*, *CB2*, and *CB1/CB2* showed increased survival reaching significance for *CB1* and *CB1/CB2* (*p* = 0.035 and 0.025, respectively). Both cannabinoid agonists inhibited the proliferation and expression of *EGFR* in lung cancer cells, and CBD potentiated the effect of THC. THC and CBD alone or in combination restored the epithelial phenotype, as evidenced by increased expression of *CDH1* and reduced expression of *CDH2* and *VIM*, as well as by fluorescence analysis of cellular cytoskeleton. Finally, both cannabinoids reduced the *in vitro* migration of the three lung cancer cells lines used.

**Conclusions:**

The expression levels of *CB1* and *CB2* have a potential use as markers of survival in patients with NSCLC. THC and CBD inhibited the proliferation and expression of *EGFR* in the lung cancer cells studied. Finally, the THC/CBD combination restored the epithelial phenotype *in vitro*.

## Introduction

Lung cancer is the leading cause of cancer-related death; more than 1 million patients are diagnosed annually. In many cases, life expectancy is only a few months and the 5-year survival rate is < 15% [1–2]. Non-small cell lung cancer (NSCLC) represents 85% of all lung cancers and the most common subtypes are adenocarcinoma and squamous cell carcinoma [3]. Both subtypes are characterized by genetic abnormalities, which lead to alterations in signaling pathways that are targets for drug therapies [4]. All patients with NSCLC eventually develop resistance to antitumor agents, including endothelial growth factor receptor (EGFR) inhibitors and chemotherapeutics, possibly due to abnormal signal transduction and *EGFR* overexpression [5–7]. This necessitates the development of new complementary pharmacological agents.

The endocannabinoid system is composed of the G-protein–coupled receptors *CB1* and *CB2*, their endogenous ligands anandamide and 2-araquidonoglicerol, and their synthetic and degradative enzymes [8]. *CB1* receptor is expressed not only in the central nervous system, but also in other tissues and organs, where its activation exerts both central and peripheral effects [9]. *CB2* is expressed in immune cells, microglia, vascular smooth muscle cells, hepatic stellate cells, and endothelial cells. *CB2* modulates Ca^2+^ channels, mitogen-activated protein kinase activation, and cAMP production [9]. According to reports, both receptors are expressed in, for example, breast and prostate cancer, glioblastoma, rhabdomyosarcoma, and colorectal cancer cells [10–14].

Although *CB1* and *CB2* are expressed in a variety of cancer cell lines and types of tumors, including adenocarcinomas [15], the relationships of their expression levels with lesion characteristics and disease progression have not been investigated. We thus assessed the correlation between the expression levels of the two receptors and the disease and clinical characteristics of a cohort of patients with NSCLC.

Cannabinoid-receptor agonists have potential as complementary pharmacological agents for NSCLC due to their analgesic, antianorexic, antiemetic and antineoplastic properties. For example, cannabinoid receptor agonists modulate key signaling pathways—including the extracellular signal-related kinase (ERK), phosphoinositide 3-kinase (PI3K), p38 mitogen-activated protein kinase (p38 MAPK), and ceramide pathways—*in vitro* and *in vivo*, inducing apoptosis and inhibiting cancer dissemination [16–18]. Cannabinoids act on cannabinoid receptors and include endocannabinoids (produced naturally in the body of animals), phytocannabinoids (found in cannabis and some other plants), and synthetic cannabinoids (manufactured artificially). *Cannabis sativa* contains more than 150 cannabinoid agonists, including Δ^9^-tetrahydricannabinol (THC), cannabidiol (CBD), cannabinol, cannabichroemene, and cannabigerol [19]. Among these, THC and CBD have demonstrated antitumor efficacy against glioblastoma, leukemia, and melanoma, as well as cervical, breast, and prostate cancer [20]. THC is a partial agonist of *CB1* and *CB2* receptors, and induces analgesia and muscle relaxation, suppresses emesis and stimulates appetite; however, the psychotropic activity of THC limits its clinical use [21]. CBD has greater affinity for *CB2* than *CB1* [22]. It also stimulates vanilloid pain receptors (VR1) and inhibits the uptake of anandamide [23]. CBD has anti-inflammatory, neuroprotective, anticonvulsant, muscle-relaxant, and anti-psychotropic effects [22]. Combined used of THC and CBD confers greater benefits, as CBD enhances the effects of THC and reduces its psychotropic activity. Thereby, in rats, CBD administered with THC ameliorate adversely effect (e.g. dysphoria) often associated with THC alone and did not alter the discriminative stimulus effect of THC [24]. Moreover, this combination enhances anticancer activity compared with THC alone and reduces the doses of THC that are needed to inhibit tumor growth [25–27]. CBD has also been shown to alleviate some of the undesired effects of THC administration, such as convulsions, discoordination and psychotic events, and, therefore, improves the tolerance of cannabis-based medicines [25]. Moreover, the combined used of THC and CBD reduces cell viability and migration, and induces apoptosis in human glioblastoma [28]; however, its effect on NSCLC is unclear. We thus investigated the influence of *CB1* on the antineoplastic effects of THC in an *in vitro* model of lung cancer.

The epithelial-to-mesenchymal transition (EMT) involves complex phenotypic changes of tumor cells [29]. During the EMT, epithelial markers (including E-cadherin) are downregulated and mesenchymal markers (such as vimentin [*VIM*], N-cadherin, and smooth muscle alpha actin [ASMA]) are upregulated by the transcription factors snail, ZEB1, and ZEB2 in a manner involving transforming growth factor-β1 (TGF-β1) [30–32]. Ravi *et al*. assessed the effect of the CB2 agonist JWH-015 on the EMT in A549 cells exposed to TGF-β1, and in an *in vivo* model of tumorigenesis [4]. They founded that JWH-015 inhibited EMTE in A549 cells and also reversed the mesenchymal nature of CALU-1 cells by downregulating *EGFR* signaling. JWH-015 decreased also migratory and invasiveness of A549 cells. In the present study, we also evaluated the effect of the non-selective cannabinoids agonists THC and CBD by separate or in combination on the EMT in three lung cancer cell lines and we explored the additive effect of CBD in combination with THC.

In this study, we investigated the correlation of the expression levels of *CB1* and *CB2* with the clinical and pathological features of 157 samples of well-characterized lung tumors. We determined the effect of the non-selective cannabinoid agonists CBD and THC by separate or in combination on cell proliferation, the expression of *EGFR*, the EMT and migration of A549, H460 and H1792 cells exposed to TGF-β or EGF. We found an additive effect of THC/CBD, which support the use of this combination in order to minimize the dose of THC and its psychotropic effects. The results emphasize the importance of the endocannabinoid system, as well as the potential use of *CB1* and *CB2* as biomarkers of survival in patients with NSCLC.

## Materials and methods

### Patients

We retrospectively analyzed 157 tumor samples from patients with NSCLC. The tumor samples were stored at −80° C. The clinical and histopathological features of the tumors are listed in Table 1. All of the subjects provided informed consent. The study was conducted in accordance with the Declaration of Helsinki and applicable local regulatory requirements and laws. The procedures were approved by the Ethics Committee of the University General Hospital of Valencia (Spain).

**Table 1:** Clinical and histopathological characteristics of the patients with NSCLC.

	N	%
**Age** (median)	61 (35–81)	
**Gender**		
Male	136	86.6
Female	21	13.4
**Smoker**		
Never	16	10.2
Ex-smoker	64	40.8
Active smoker	77	49.0
**Stage**		
IA	13	8.3
IB	62	39.5
IIA	5	3.2
IIB	29	18.5
IIIA	32	20.4
IIIB	12	7.6
IV	4	2.5
**TNM**		
T1	21	13.4
T2	88	56.1
T3	32	20.4
T4	16	10.2
N0	89	56.7
N1	21	13.4
N2	25	15.9
Nx	2	1.3
M0	151	96.2
M1	6	3.8
**Grade**		
Well-differentiated	23	14.6
Moderately differentiated	83	52.9
Poorly differentiated	51	32.5
**Histology**		
Epidermoid	79	50.3
Adenocarcinoma	57	36.3
Giant cells	5	3.2
Adenosquamous	6	3.8
Carcinoid	1	0.6
Microcytic	1	0.6
Non-differentiated	6	3.8
Neuroendocrine	2	1.3

### Cannabinoids

THC and CBD were supplied by Sigma-Aldrich (Madrid, Spain). They were prepared at 150 mM amounts in ethanol and used individually or mixed 1:1 at concentrations of 10–100 μM.

### Cells and *in vitro* experiments

A549, H460 and H1792 human lung cancer cells were purchased from the American Type Culture Collection (Rockville, MD, USA). The cells were growth in 25 cm^2^ culture flasks in Roswell Park Memorial Institute (RPMI) medium containing endotoxin-free fetal bovine serum (FBS, 5% for A549 and H469 cells, 10% for H1792 cells), L-glutamine (4 mM), penicillin (100 U/mL), streptomycin (10 μg/ml) and amphotericin B (0.25 μg/mL).

To assess the antitumor effect of THC and CBD, cells were exposed to 10–100 μM THC or CBD, individually or in combination (1:1 ratio) for 48 hours. Cell proliferation and the expression levels of *CB1*, *CB2*, and *EGFR* were evaluated. To analyze the effect of THC/CBD on the EMT, A549, H460 and H1792 cells were stimulated with TGF-β (15 ng/ml) in the presence or absence of 30 μM THC or 30 μM CBD alone or in combined at 10 μM for 48 hours. The effects on the cytoskeleton and the expression levels of *CDH1*, *CDH2* and *VIM* were evaluated. Inhibition of the migration of the three cell lines in the presence of 20 ng/ml EGF was also evaluated using the scratch wound assay method.

### Cell proliferation assay

The proliferation of A549, H460 and H1792 cells was evaluated by enzyme immunoassay for bromodeoxyuridine (BrdU) incorporation (BrdU Cell Proliferation Assay Kit; Merck Millipore, Darmstadt, Germany). Cells were seeded in 96-well culture plates at 5,000 cells/well and cultured for 24 hours in culture medium as described above. The cells were then cultured for 48 hours in BrdU-containing medium in the presence or absence of 10–100 μM THC and CBD, individually or in combination (1:1 ratio). Culture medium was removed, the cells were fixed, and BrdU incorporation was evaluated with an anti-BrdU antibody by determining the absorbance at 450/550 nm following the manufacturer’s instructions.

### Determination of *CB1*, *CB2*, *EGFR*, *CDH1*, *CDH2*, and *VIM* expression levels

Total RNA was extracted from freshly frozen tumor samples and from cell cultures using the TRIzol reagent (Thermo Fischer Scientific Inc., Waltham, MA, USA) according to the manufacturer’s instructions. The RNA concentration was determined by spectrophotometry using a Nanodrop 2000 spectrophotometer (Fischer Scientific, Madrid, Spain). Only extracts with a 260/280 nm ratio > 1.8 were used. RNA integrity was evaluated by capillary electrophoresis using a Bioanalyzer (Agilent Technologies, Santa Clara, CA, USA). Only extracts with a RIN of ~10 were used for the determination of gene expression levels.

Random hexamers were used to synthesize complementary DNA (cDNA) using TaqMan RT reagents (Applied Biosystems, Foster City, CA, USA) following the manufacturer’s instructions. Gene expression levels were assayed by reverse transcriptase-polymerase chain reaction (RT-PCR) using Assays on Demand for *CB1*, *CB2*, *EGFR*, *CDH1*, *CDH2*, and *VIM* (Applied Biosystems Madrid, Spain). Reactions were carried out in a 7900HT Real-Time Thermocycler (Applied Biosystems, Madrid, Spain). The comparative ΔΔCt method with glyceraldehyde 3-phosphate dehydrogenase (*GAPDH)* as an endogenous control was used to calculate the relative gene expression levels [33]. For human tissue samples *glucuronidase beta* (*GUSB*) gene was used as endogenous control.

### Fluorescence staining of F-actin

Filamentous actin (F-actin) in lung cancer cell lines was visualized using rhodamine-conjugated phalloidin (Molecular Probes, Thermo Fisher Scientific, Waltham, MA, USA). Cells were cultured on slides to sub-confluence, and exposed to TGF-β (15 ng/mL) in the presence or absence of 10 μM THC/CBD, which was added 2 hours before TGF-β addition. Control cells received neither TGF-β nor THC/CBD. Forty-eight hours later, the cells were washed twice with pre-warmed phosphate-buffered saline, pH 7.4 (PBS) and fixed in 3.7% formaldehyde in PBS for 10 minutes at room temperature. Next, the cells were permeabilized with 0.1% Triton X-100 in PBS for 3 to 5 minutes. To reduce non-specific background, the samples were pre-incubated with PBS containing 1% bovine serum albumin (BSA) for 20–30 minutes. Next, 5 μL of phalloidin methanolic stock solution were diluted in 200 μL PBS for each sample, and the mixture was added to the samples and incubated for 20 minutes. Finally, the samples were washed, the nuclei were stained with 4',6-diamidino-2-phenylindole, and the samples were visualized under a DM2500 fluorescence microscope (Leica, Wetzlar, Germany).

### Wound healing assay

The wound healing assay was performed as previously described [34]. A549, H460 and H1792 cells were cultured to confluence in six-well culture plates. Then, cultures were scratched to produce a ‘wound’ using sterile 10 μl pipette tips. Cell debris was removed from the culture by washing with sterile PBS for three times. The cells were then cultured in the presence or absence of THC or CBD alone or in combination in serum-free culture medium with 10 ng/ml *EGF* for 48 hours. Images were recorded using a phase contrast photomicroscope (Nikon), and cell migration was quantified with respect to the control (scratched cultures, non-exposed to *EGF* cells) using Scion Image software (Alpha 4.0.3.2).

### Data analysis

Comparisons of categorical variables were conducted using the non-parametric Mann–Whitney U test or Kruskal–Wallis test. Survival was analyzed by the Kaplan–Meier method and differences between groups were assessed by log-rank test, performed using SPSS software (IBM Corp., Armonk, NY). A *p*-value of < 0.05 was considered indicative of statistical significance.

*In vitro* data are shown as means ± SD and were subjected to analysis of variance (ANOVA) followed by Tukey´s multiple-comparison test (GraphPad Software Inc., San Diego, CA, USA). Significance was accepted at *p* < 0.05. The inhibitory concentration 50 (IC_50_) of THC and CBD, alone or in combination, was calculated according to proliferation data using the Graphpad software.

## Results

### *CB1* and *CB2* overexpression is associated with prolonged survival

First, we evaluated the expression levels of *CB1* and *CB2* in a well-characterized cohort of patients with NSCLC. The clinical and histopathological features of the patients are summarized in Table 1. The expression levels (ΔCt) of *CB1* and *CB2* were calculated relative to that of the house-keeping gene *glucuronidase beta* (*GUSB*). Multivariate analysis revealed no significant associations between the relative expression levels of *CB1* and *CB2* and the following clinical characteristics: gender (*p* = 0.057 and *p* = 0.267, respectively), progression (*p* = 0.159, *p* = 0.209), age (*p* = 0.690, *p* = 0.835), smoking (*p* = 0.223, *p* = 0.512), stage (*p* = 0.317, *p* = 0.961) and histology (*p* = 0.650, *p* = 0.550). There was a significant association between chronic obstructive pulmonary disease (COPD) and the expression of *CB2* (*p* = 0.020) but not that of *CB1* (*p* = 0.758).

**Fig 1:**
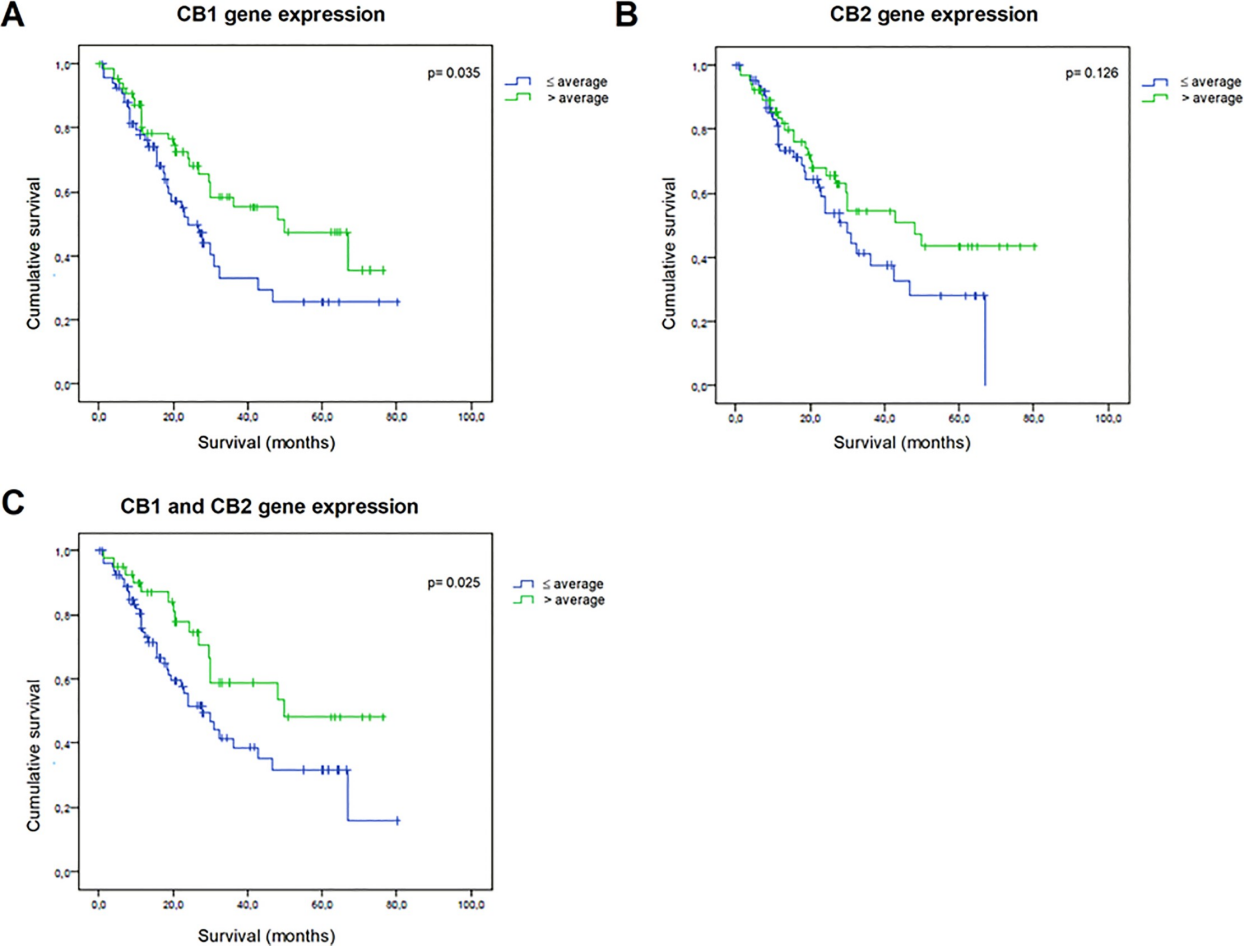
Cannabinoid receptors (*CB1* and *CB2*) expression in 157 patients with non-small cell lung cancer (NSCLC). Analysis of the survival of patients with NSCLC according to whether their *CB1* (A), *CB2* (B), or *CB1/CB2* (C) ΔCt values were higher or lower than the mean ΔCt value of the entire cohort.

A survival analysis was performed according to the ΔCt values of *CB1* and *CB2* (Fig 1). Patients with a ΔCt value of *CB1*, *CB2*, or *CB1* and *CB2* equal to or greater than the mean ΔCt value of the cohort, had longer survival than those with ΔCt values lower than the mean cohort (*p* = 0.035, 0.126, and 0.025, respectively).

### THC/CBD inhibited the proliferation and *EGFR* expression in lung cancer cells

Next, we assessed the anti-proliferative effects of THC and CBD on A549, H460 and H1792 cells *in vitro*. The expression of *CB1* was significantly higher than that of *CB2* (6.58 ± 1.29-fold, n = 6, *p* = 0.0136; 3.15 ± 1.07-fold, n = 6, *p* = 0.0160; and 2.15 ± 0.45-fold, n = 6, *p* = 0.00247, for A549, H460 and H1792 cells, respectively). Because of the cytotoxic effects of both compounds at >100 μM, detected by 3-(4,5-dimethylthiazol-2-yl)-2,5-diphenyltetrazolium bromide (MTT) assay, THC and CBD concentrations in the range 10–100 μM were used.

**Fig 2:**
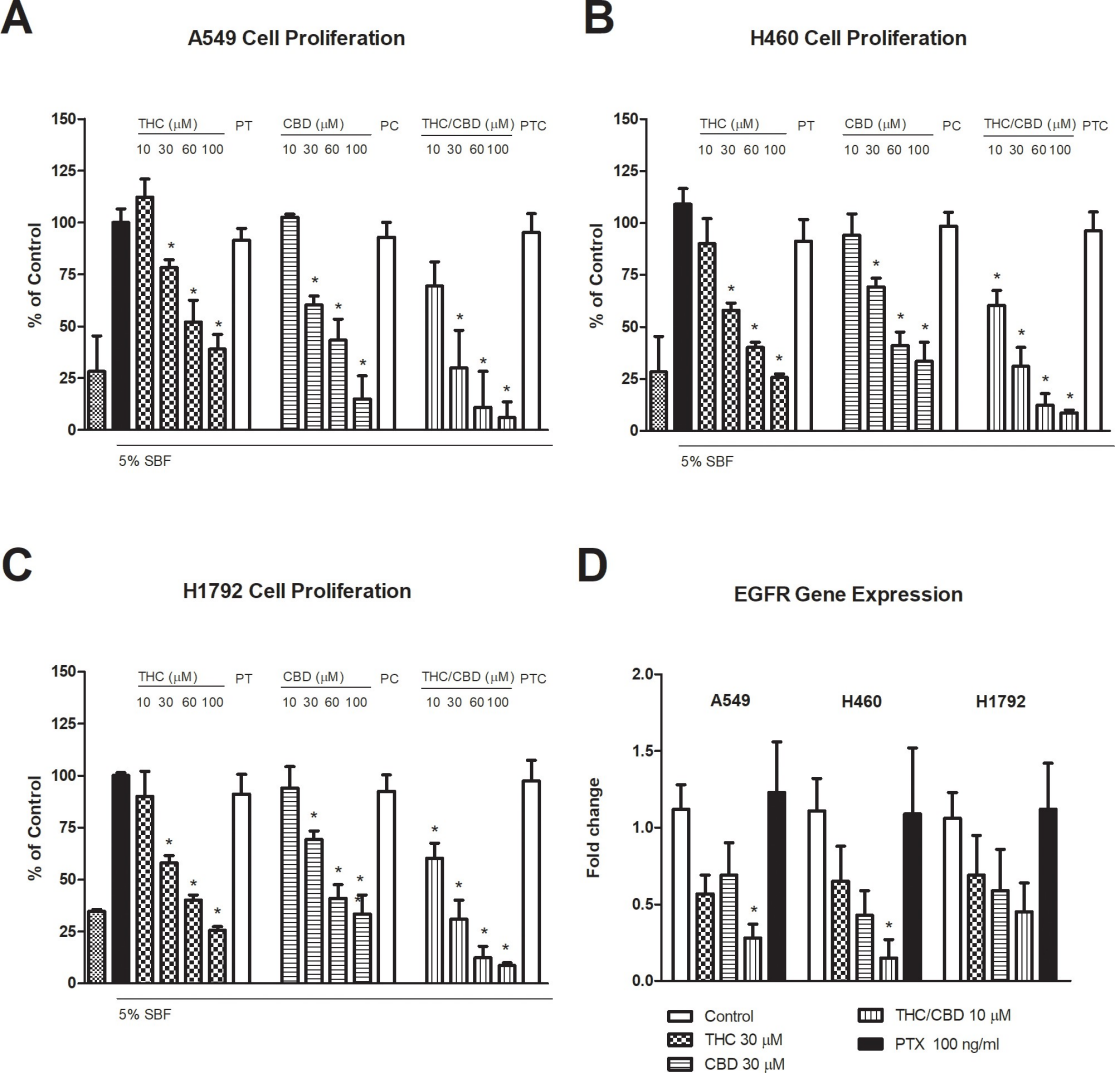
Tetrahydrocannabinol (THC) and cannabidiol (CBD) inhibit the proliferation and epithelial growth factor receptor (*EGFR*) expression in lung cancer cells. Proliferation of A549 (A), H460 (B) or H1792 (C) cells treated with 10–100 μM THC, CBD, or CBD/THC. PT (100 μM THC in the presence of PTX 100 ng/ml). PC (100 μM CBD in the presence of PTX 100 ng/ml). PTC (100 μM THC/CBD in the presence of PTX 100 ng/ml). *EGFR* expression in cells treated with 30 μM THC or CBD, or 10 μM THC/CBD in the presence or absence of 100 ng/mL PTX (D). The results are expressed as mean ± SD of three independent experiments. Each condition was evaluated in six replicates from three independent wells. # *p* < 0.05 *versus* the control group.

THC and CBD separately or in combination significantly inhibited the proliferation of A549 cells in a dose-dependent fashion (Fig 2A). Concentrations of 10–100 μM of both cannabinoid agonists were studied. IC_50_ of 27.25 and 37.31 μM were calculated for THC and CBD, respectively. The combination of both cannabinoids significantly reduces the IC_50_ to 12.94 μM, demonstrating an additive effect. Similar results were founded for H469 cells (IC_50_ of 30.64, 39.78 and 8.04 μM for THC, CBD or THC/CBD, respectively, Fig 2B) and for H1792 cells (IC_50_ of 33.39, 46.41 and 14.55 μM for THC, CBD or THC/CBD respectively, Fig 2C). In all cases, the anti-proliferative effect of THC/CBD was inhibited by pertussis toxin (PTX) at 100 ng/mL. According to the calculated IC_50_ values, a dose of 30 μM for THC and for CBD alone, and 10 μM for THC combined with CBD were used for subsequent experiments.

Both cannabinoid agonists inhibited the *EGFR* expression in A549, H460 and H1792 cells as it is shown in Fig 2D. Only the combination of THC and CBD significantly decreased *EGFR* expression in A549 and H460 cells, which was inhibited by PTX at 100 ng/mL.

### THC/CBD inhibits the EMT in lung cancer cells

**Fig 3:**
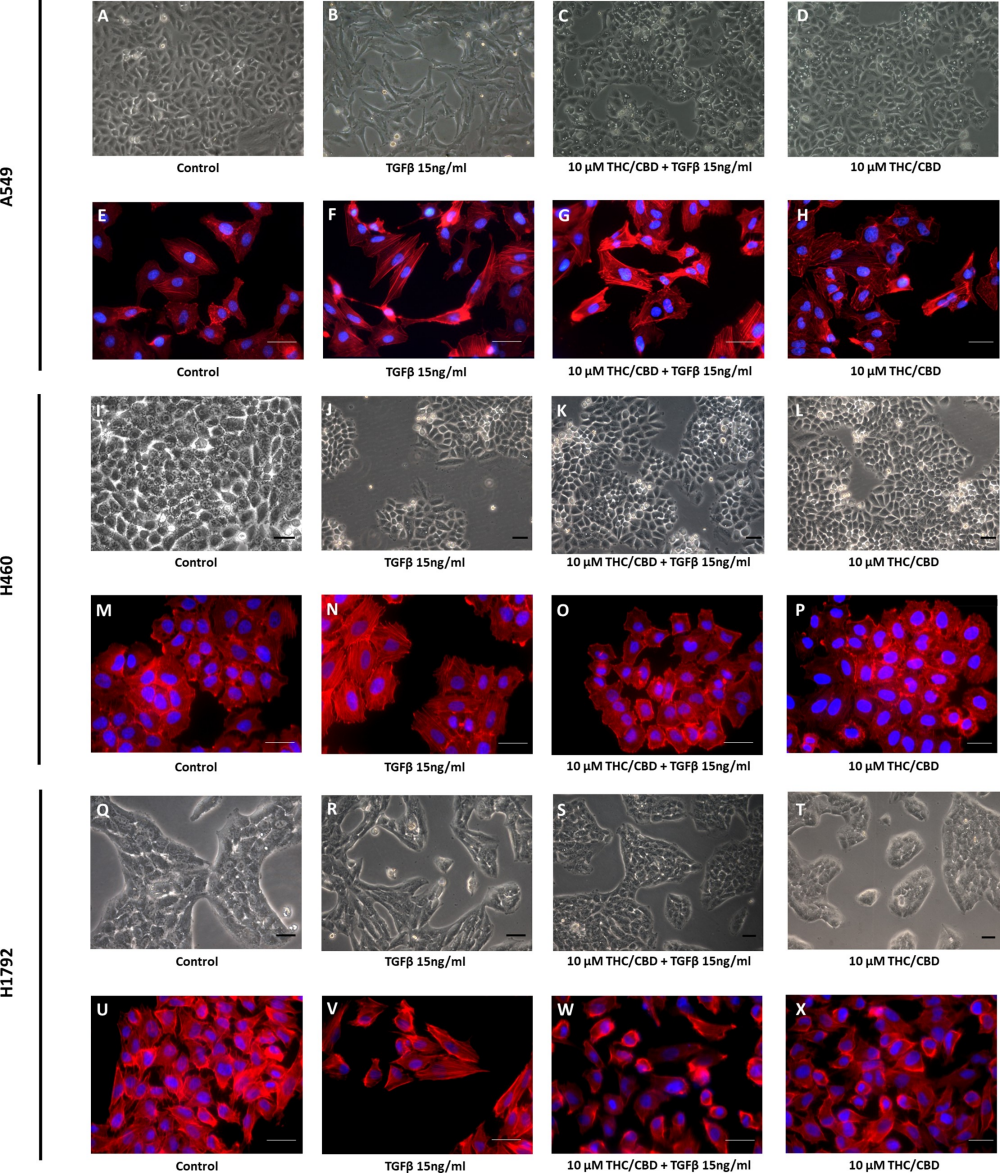
THC/CBD inhibits the epithelial-to-mesenchymal transition (EMT) in cancer cells. A549 (A-H), H460 (I-P) and H1792 (Q-X) cells were cultured in the presence or absence of 10 μM THC/CBD, and/or 15 ng/mL TGF-β. Representative images of cell morphology and fluorescence images of F-actin are shown. All experiments were performed in six replicates and five fields were assessed per condition. Scale bars equal to 25 μm.

A549, H460 and H1792 cells stimulated with TGF-β were used to assess the effect of THC/CBD on the EMT [4, 31, 32]. Cells were stimulated with 15 ng/ml TGF-β in the presence or absence of 10 μm THC/CBD for 48 hours. Morphological changes were evaluated by phase-contrast light microscopy. Representative results are observed in the Fig 3. Cells exposed to 15 ng /ml TGF-β (Fig 3B, 3J and 3R) became more loosely and acquired a spindle-shaped morphology compared to control cells (Fig 3A, 3I and 3Q). TGF-β-exposed cells treated with 10 μm THC/CBD suppressed these changes (Fig 3C, 3K and 3S). No effect of 10 μM THC/CBD was observed in cells not exposed to TGF-β1 (Fig 3D, 3L and 3T). These effects were found for the three cancer cells included in these experiments. Nevertheless, they were more evident for A549 and H1792 cells than for H460 cells. Changes in cell morphology corresponded to the reorganization of F-actin. In control cells non-exposed to TGF-β or to THC/CBD, phalloidin-labelled F-actin was organized into cortical bundles tightly associated with cell-cell adhesions (Fig 3E, 3M and 3U). After incubation with TGF-β for 48 hours, F-actin was assembled into thicker parallel bundles (actin stress fibers) that crossed the cell surface (Fig 3F, 3N and 3V). These changes were reversed in cultures treated with 10 μM THC/CBD (Fig 3G, 3O and 3W). Interestingly, A549 cells treated with THC/CBD in the absence of TGF-β showed enhanced cell-cell adhesion and thinner actin bundles than control cells (Fig 3H). This effect was not observed for H460 or H1792 cells, in which no *per se* effect of 10 μM THC/CBD was observed (Fig 3P and 3X).

**Fig 4:**
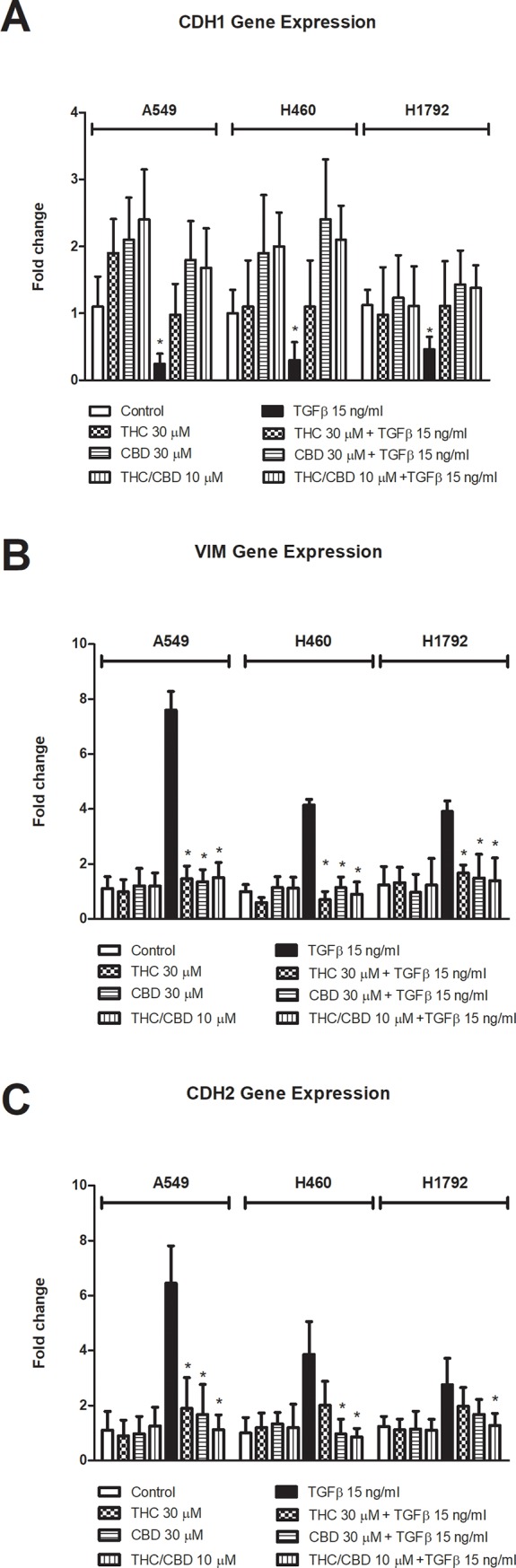
Tetrahydrocannabinol (THC) and cannabidiol (CBD) inhibit gene expression of EMT-related genes. A549, H460 and H1792 cells were treated with 30 μM THC or CBD, or 10 μM THC/CBD and/or 15 ng/ml TGF-β. Relative gene expression levels of *CDH1* (A), *VIM* (B) and *CDH2* (C) were calculated by real-time RT-PCR using *GAPDH* as the endogenous control. The results are means ± SD of three independent experiments. Each condition was evaluated in six replicates. The comparative ΔΔCt method was used to analyze the data. #p < 0.05 *versus* the control group.

The changes observed in cell morphology were consistent with those of the expression of the EMT markers *CDH1*, *CDH2* and *VIM* (Fig 4A–4C). The expression of *CDH1* was significantly downregulated (0.25 ± 0.15, 0.30 ± 0.27 and 0.46 ± 0.19-fold for A549, H460 and H1792 cells, respectively), while that of *CDH2* (6.45 ± 1.36, 3.86 ± 1.20 and 2.75 ± 0.97-fold for A549, H460 and H1792 cells, respectively) and *VIM* (7.59 ± 0.69, 4.15 ± 0.20 and 3.91 ± 0.39-fold for A549, H460 and H1792 cells, respectively) were upregulated in cells stimulated with TGF-β compared to control cells. These changes were significantly reversed by CBD or THC alone (30 μM) or in combination (10 μM), which restored the expression values close to those found in treated with CBD or THC but non TGF-β-stimulated cells. The relative expression tendencies found in the three cell types included in this investigation were similar.

### Cannabinoids inhibit EGF-induced cell motility in cancer cells

**Fig 5:**
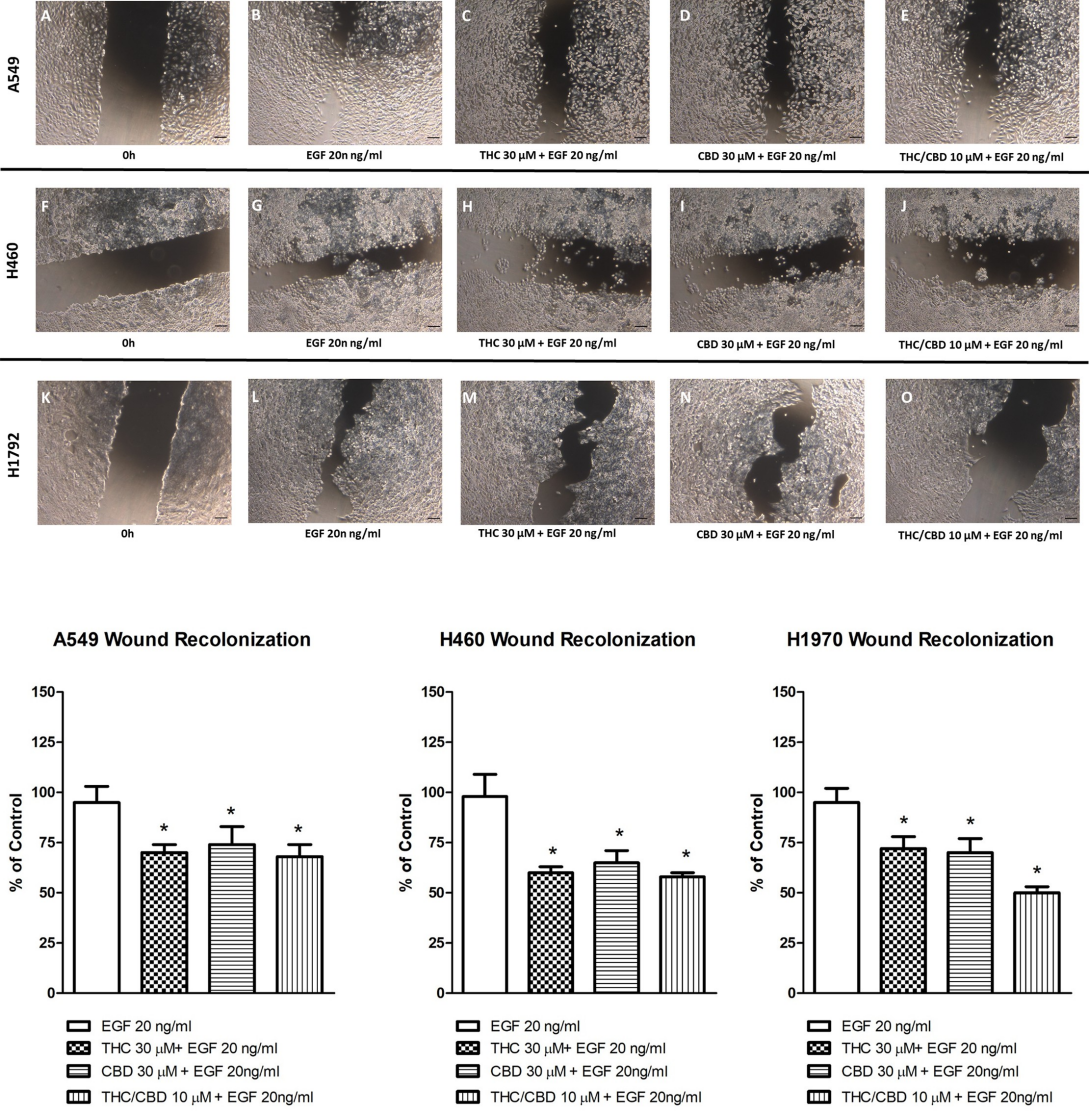
Tetrahydrocannabinol (THC) and cannabidiol (CBD) inhibit EGF-induced cell migration in cancer cells. A549, H460 and H1792 cells were cultured until confluence. Scratching was performed and the cells were cultured in the presence or absence of 30 μM THC or CBD, or 10 μM THC/CBD. Twenty ng/ml EGF was used to induce cell migration. Cells were cultured for 48 hours. Phase-contrast images were collected of 5 fields from each well. Representative images are shown. Total wound areas were measured using Scion Image software, and the percentage of wound recolonization was calculated. The results are means ± SD of three independent experiments. #*p* < 0.05 *versus* the control group. Scale bars equal to 50 μm.

Finally, A549, H460 and H1792 cells were exposed to 20 ng/ml EGF in order to analyze cell migration, as previously reported [34]. As summarized in Fig 5, EGF induces cell migration in the three types of cancer cell used. Both THC and CBD inhibited cell motility, separately or in combination. Although discrete differences were found when comparing data from each cell type, no significant differences were found between them. Inhibition of both cannabinoids tested was close to 30% compared to untreated cells stimulated with EGF.

## Discussion

NSCLC is the leading cause of cancer-related mortality in the United States [1–2]. It is an aggressive disease strongly associated with smoking that frequently disseminates; thus, patients are commonly diagnosed with metastatic lesions. Early trials established that radiotherapy was more effective than surgery [35], but due to the extremely low survival rates, chemotherapy has emerged as the optimal modality of treatment. NSCLC is characterized by overactivity of *EGFR*, which could explain the limited efficacy of chemotherapy [36]. Regrettably, the use of selective *EGFR* inhibitors or monoclonal antibodies against *EGFR* has failed, so other therapies for NSCLC are under investigation [36]. Among them, the use of cannabinoids is being evaluated using *in vivo* and *in vitro* models of breast, prostate, and lung cancer [37]. Cannabinoids activate the specific G-protein coupled receptors *CB1* and *CB2*. Although *CB1* expression has been associated with the brain, and that of *CB2* with the immune system, both receptors are expressed to some degree in other cells and organs (*e*.*g*., placenta, liver, endothelial cells, smooth muscle cells, and kidney). Although the expression of *CB1* and *CB2* in NSCLC has been evaluated, to our knowledge, no study has explored the correlation between their expression levels and the clinical and histopathological features of patients with NSCLC. In this study, only COPD was significantly associated with the expression of *CB2* (*p* = 0.020), and non-significantly with that of *CB1* (*p* = 0.758). *CB2* is reported to be related to smoking; for example, nicotine addiction is inhibited by *CB2* antagonists in mice [38] and the level of *CB2* mRNA in blood increases after cessation of marijuana smoking [39]. As smoking is the main causative agent of COPD, further studies of this association are needed to enhance our understanding of the relationship between COPD and lung cancer [40].

The few studies of the prognostic value of *CB* expression have reported discrepant results [41]. In tumors of the nervous system, there is a relationship between high expression of *CB1* and *CB2* with astrocytoma, and of *CB2* with glioblastoma and malignancy [42–43]. In contrast, high expression of *CB2* is associated with an increase of macrophage invasion of brain tumors [44]. In fact, high expression of *CB1* is associated with increased severity of prostate and colorectal cancer [45–46]. In contrast, high expression of *CB1* and *CB2* indicates longer disease-free survival in patients with hepatocellular carcinoma [47], while a lower expression of *CB1* is correlated with a lower survival in patients with pancreatic ductal carcinoma [48]. In the present study, NSCLC patients with high expression of *CB1* and *CB2* showed prolonged survival, which supports their potential use as biomarkers.

Cannabinoids inhibit the proliferation of breast, prostate, and bone cancer cells [31]. However, little is known about the beneficial effects of THC/CBD. Our results indicate that THC and CBD inhibit cell proliferation in a dose-dependent manner. Moreover, CBD enhances the antiproliferative effect of THC in A549, H460 and H1792 cells, in agreement with previous reports [24–27]. CBD and THC also reduce *EGFR* expression in the three types of cells analyzed in this study. Both cannabinoids are known to inhibit the *EGFR* pathway, which modulates the proliferation of tumor cells [49–50].

Lung cancer has a propensity to disseminate and invade other tissues [49]. THC inhibits *EGFR*-induced migration of A549 cells and subcutaneous metastasis in mice with severe combined immunodeficiency [50]. Our results concerning cell migration are in line with those previously reported. Both cannabinoid agonists inhibited EGF-mediated cell migration in A549, H460 and H1792 cells. CBD has an additive effect on the inhibition of THC-mediated cell migration, which supports the beneficial use of both cannabinoids in combination. The metastasis of epithelial tumors involves a series of phenotypic changes, known as the EMT. Cannabinoids have been reported to inhibit EMT in gastric, endometrial and NSCLC cancer cells [4, 34,51]. However, this important property of cannabinoids has not been extensively investigated. For this reason, we explored the effect of THC/CBD on EMT in cancer cells treated with TGF-β *in vitro*. The combination of THC/CBD strongly affects the cytoskeletal and molecular changes characteristics of EMT, including the downregulation of *CDH1* and the upregulation of *CDH2* and *VIM*. Interestingly, in A549 cells, in the absence of TGF-β, exposed to CBD and THC, increased cell-cell adhesion and reduced thickness of actin bundles compared to control cells was observed. These effects could be explained by the mesenchymal phenotype of A549 cells, which are of epithelial origin; indeed, these cells produce TGF-β [52]. Therefore, THC and CBD suppress the basal EMT phenotype, which enhances the medical importance of cannabinoids.

In summary, our results indicate that *CB1* and *CB2* expression levels have potential as biomarkers for the survival of patients with NSCLC, and that THC and CBD could be used to suppress cell proliferation and EMT. Moreover, the combined use of both compounds could be of interest due to the additive effects observed and could minimize the undesired effects of THC.
